# Profiling of the serum MiRNAome in pediatric egyptian patients with wilms tumor

**DOI:** 10.3389/fmolb.2024.1453562

**Published:** 2024-10-15

**Authors:** Fatma S. Mohamed, Deena Jalal, Youssef M. Fadel, Samir F. El-Mashtoly, Wael Z. Khaled, Ahmed A. Sayed, Mohamed A. Ghazy

**Affiliations:** ^1^ Biotechnology Program, Institute of Basic and Applied Science, Egypt-Japan University of Science and Technology, Alexandria, Egypt; ^2^ Biochemistry Program, Faculty of Science, Minia University, Minia, Egypt; ^3^ Genomics and Epigenomics Program, Department of Basic Research, Children’s Cancer Hospital Egypt, Cairo, Egypt; ^4^ Bioinformatics Group, Center for Informatics Science, School of Information Technology and Computer Science, Nile University, Giza, Egypt; ^5^ Leibniz Institute of Photonic Technology, Jena, Germany; ^6^ Department of Pediatric Oncology, National Cancer Institute, Cairo University, Cairo, Egypt; ^7^ Department of Pediatric Oncology, Children’s Cancer Hospital Egypt, Cairo, Egypt; ^8^ Department of Biochemistry, Faculty of Science, Ain Shams University, Cairo, Egypt

**Keywords:** next-generation sequencing, miRNAome profiling, blood-based biomarkers, wilms tumor, histopathological subtyping

## Abstract

Wilms tumor (WT) is a pediatric kidney cancer associated with poor outcomes in patients with unfavorable histological features such as anaplasia. Small non-coding RNAs, such as miRNAs, are known to be involved in WT pathogenesis. However, research on the clinical potential of blood-based miRNAs is limited. This study aimed to profile aberrantly expressed miRNAs in WT serum samples, evaluate their potential to differentiate standard-risk patients with favorable histology from those with anaplastic WTs, and assess their clinical value as minimally invasive biomarkers for WT detection. The study used next-generation sequencing (NGS) to analyze miRNA expressions in serum samples from 37 Egyptian children, including 10 healthy individuals, 14 with non-anaplastic WTs (favorable histology FH-WTs), and 13 with anaplastic WTs (unfavorable histology UnFH-WTs). Functional enrichment analysis was conducted to identify critical pathways and biological processes affected by dysregulated miRNAs, and a network was created for the most promising miRNA-target interactions linked to WT. The study identified a distinct miRNA expression signature of 45 miRNAs (3 upregulated and 42 downregulated) in WT serum samples compared to healthy controls, with 29 miRNAs exclusively dysregulated in FH-WTs and 6 miRNAs dysregulated solely in UnFH-WTs. These dysregulated miRNAs displayed significant enrichment in cancer-related pathways, such as PI3K/AKT, FOXO, and MAPK signaling. In relation to WT clinicopathological features, decreased levels of hsa-miR-2355-3p showed a significant positive correlation with clinical stage (*r* = 0.6597, *p* = 0.0006) and WT metastasis (*r* = 0.439, *p* = 0.021). The ROC curve analysis revealed that multiple dysregulated miRNAs in WT, specifically hsa-miR-7-5p, hsa-miR-146a-5p, hsa-miR-378a-3p, and hsa-miR-483-5p, exhibited high diagnostic potential for WT, with AUC values exceeding 0.86. Among WT histopathology types, the hsa-miR-1180-3p showed a 2.3 log2fold difference in expression between UnFH-WTs and FH-WTs, indicating its potential as a biomarker with 92% sensitivity and 85% specificity for identifying UnFH-WTs. Its target genes were enriched in pathways related to cell division and cell cycle regulation. In conclusion, hsa-miR-1180-3p could be a reliable blood-based biomarker for distinguishing WT histopathological types, and further research is needed to validate its clinical value.

## Introduction

Wilms tumor (WT), also known as nephroblastoma, is a common renal malignancy in childhood, and its pathogenesis involves diverse genetic and epigenetic alterations (Pater et al., 2021)., WTs are histopathologically classified into two main categories according to the Children Oncology Group (COG): anaplastic WTs with unfavorable histological features (such as focal and diffuse anaplasia) and non-anaplastic WTs with favorable histology and no presence of anaplasia (Spreafico et al., 2021). The prognosis of the tumors is significantly influenced by their histopathological type (Nelson et al., 2021). Despite high survival rates, patients with unfavorable histology still exhibit poor outcomes and are at higher risk of kidney failure, disease recurrence, and death (Bhutani et al., 2021).

The most observed genetic alterations in sporadic WTs include mutations in *WTX, WT1, CTNNB1, and TP53*, along with 11p15 imprinting abnormalities. However, these changes only account for approximately one-third of WT cases (Treger et al., 2019). Recently, molecular profiling and whole-exome sequencing have identified novel genetic mutations in WT, including those in microRNA processing genes *DROSHA, DGCR8,* and *DICER1*. Mutations in these genes can impair tumor-suppressing miRNAs, such as the let-7 family, which regulate oncogenes *MYCN* and *LIN28* in WT (Rakheja et al., 2014; Vardapour et al., 2022).

MicroRNAs (miRNAs), small noncoding RNAs with 20–22 long nucleotides, significantly control post-transcriptional gene regulation by binding to target mRNAs and either promoting their degradation or inhibiting translation. They are involved in biological processes, such as development, cell differentiation, and immune response (Saliminejad et al., 2019). Dysregulation of miRNAs has been linked to various diseases, including cancer; thus, they hold potential as both clinical biomarkers and therapeutic targets (Kabekkodu et al., 2020). Deficiencies in miRNA processing genes were found to affect miRNA biogenesis and reprogramme miRNA expressions in WT (Rakheja et al., 2014; Vardapour et al., 2022). In this context, previous studies have found aberrant miRNA expressions in both cell lines and clinical samples of WT using TaqMan Low-Density Array (TLDA), microarray and qRT-PCR profiling assays (Wang et al., 2010; Watson et al., 2013; Pérez-Linares et al., 2020). Wang et al. (2010) found miRNA expression differences in WTs vs. healthy embryonic kidney cells, while Watson et al. (2013) explored miRNA expressions and chemoresistance in high-risk blastemal WTs. Most of these studies have focused on analyzing aberrant miRNA expressions in WT tumor biopsies, with limited exploration into the clinical value of circulating miRNAs in predicting prognosis and histological subtyping. Over the past decade, researchers have focused on developing non-invasive biomarkers or liquid biopsies for various clinical purposes, and the stability of miRNA expressions in biofluids makes them promising non-invasive biomarkers (Andersen and Tost, 2020).The current study comprehensively profiled miRNA expression patterns in the serum of WT patients using NGS to explore their potential to discriminate standard-risk patients with favorable histology from those with high-risk features, such as anaplasia and assess their potential as minimally invasive biomarkers for WT detection. The study also evaluated key pathways and biological processes affected by these dysregulated miRNAs, providing insights into the underlying mechanisms driving tumorigenesis in WT.

## Materials and methods

### Patients and blood sample collection

The current study involved analyzing serum samples from 37 Egyptian children aged 1–8 years, including 10 healthy individuals, 14 having FH-WTs and 13 having UnFH-WTs). No congenital anomalies associated with WT were found in the study subjects. WT serum samples were obtained from the Biorepository at Children Cancer Hospital Egypt (CCHE), 57,357. These samples were collected from newly diagnosed patients at different disease stages before surgery and before receiving preoperative chemotherapy between 2022 and 2023. Histopathological assessment and staging of WT were determined following the pathology protocol of COG. The clinicopathological features of WT patients are detailed in (Supplementary Table S1). The study excluded patients with other renal cancers, kidney diseases, or any childhood malignancy. As a control group, 10 blood samples were obtained from age-matched healthy children during their periodic examination at the Borg El Arab Central Hospital, New Borg EL Arab City, Alexandria, Egypt. Children suffering from severe diseases, diabetes, or viral infections were excluded from the study. The present study has received approval from the Renal Study Team Meeting (STM), the Scientific Medical Advisory Committee (SMAC), and the Institutional Review Board (IRB) of CCHE, 57,357 on 16 March 2023. Parents or legal guardians of healthy study participants have provided a written informed consent before enrollment in the study.

### RNA isolation and quality assessment

All serum samples were treated with the miRNeasy Mini Kit (Qiagen, catalog number 217004) to extract the total RNA enriched for small RNAs according to the manufacturer’s instructions. The purity and quantity of purified RNA were evaluated using a NanoDrop™ One Spectrophotometer (NanoDrop Technologies; Thermo Fisher Scientific, Inc.). Furthermore, the integrity and size distribution of the extracted small RNAs were determined using the Agilent 2,100 Bioanalyzer (Agilent Technologies, Inc., United States).

### Library preparation and small RNA-sequencing

Barcoded cDNA libraries were generated from the purified small RNA transcripts using the NEBNext Multiplex Small RNA Library Prep Set 1 for Illumina (New England BioLabs. Inc., United States). Following library preparation, PCR products were purified using QIAquick®PCR Purification Kit (Qiagen, Hilden, Germany), and their quality was assessed on the Bioanalyzer 2,100 (Agilent Technologies, Germany) using the DNA High Sensitivity Kit (Agilent Technologies, Germany). The purified cDNA libraries were size selected using a 6% polyacrylamide gel and cleaned up using the AMPure bead clean-up protocol. The bands of 140 and 150 nucleotides correspond to adapter-ligated transcripts derived from 21 to 30 nucleotide RNA segments, mainly miRNAs and piRNAs. The size and purity of size-selected cDNA libraries were ultimately assessed on the LabChip GX Touch 24 Nucleic Acid Analyzer (PerkinElmer, United States), using the DNA NGS 3K kit (PerkinElmer, United States). The final concentration of each library was measured on the Qubit Fluorometer using the Qubit DNA high-sensitivity assay kit (Invitrogen).

Finally, the libraries were pooled in equimolar amounts and subjected to the Illumina sequencing pipeline, which involved clonal cluster generation on a flow cell with 75-bp single-end reads and sequencing-by-synthesis on the Illumina MiSeq sequencer (Illumina Inc. United States) using MiSeq Reagent Kit v3 (Illumina Inc. United States).

### Bioinformatics analysis

Raw sequencing data was quality checked using FastQC (Andrews, 2010), and adapter sequences were trimmed using Cutadapt tool (Martin, 2011). The results were compiled using MultiQC (Ewels et al., 2016). The quality-controlled reads underwent alignment to the human reference genome (GRCh38) using Bowtie 2 tool (Langmead and Salzberg, 2012, p. 2), allowing for up to two sequence mismatches. Subsequently, these reads were mapped to the RNAcentral database (release 23) (The RNAcentral Consortium, 2019). Within this database, miRbase served as a reference for miRNAs, piRbase for piRNAs, and Genecard for other small RNAs. The resulting bam files containing sncRNAs were then quantified for each sample using FeatureCounts (Liao et al., 2014). We applied specific parameters in FeatureCounts, including--minOverlap 15, --fracOverlap 0.95, and--fracOverlapFeature 0.95, to ensure accurate alignment with sncRNA annotations provided in the GFF3 file downloaded from RNAcentral.

The filtered read counts were input into the R/Bioconductor DESeq2 package (Love et al., 2014) to identify differential expression of miRNAs between WT patients and healthy controls. The significance of the differential expression was based on an absolute log2 fold change (log2FC ≥ 1 or ≤ -1) and adjusted p-value of ≤0.05. The ggplot R package (Wickham, 2016) was used to generate volcano plots from miRNA matrices in WTs, and to visualize differentially expressed miRNAs (DEmiRNAs) in lollipop plots. Heatmaps were generated from the normalized read count of DEmiRNAs using the complex Heatmap R package.

### Pathway and functional enrichment analyses

Reactome pathway (Gillespie et al., 2022) and Gene Ontology (GO) (Gene Ontology Consortium, 2004) enrichment analyses of DEmiRNAs in WT were conducted using the DIANA-miRPath v4.0 platform (Tastsoglou et al., 2023), a server designed for target-based analysis of miRNA functions. Experimentally validated miRNA-target interactions were sourced from the MiRTarBase database 2022 (Huang et al., 2022) and filtered based on strong interaction confidence annotation. The enriched terms were ranked based on p-value and a false discovery rate (FDR) threshold of <0.05.

### MiRNA-target interaction network analysis

A network for miRNA-target interactions was created using the online tool MIENTURNET (Licursi et al., 2019) to identify the miRNA-target interactions involved in WT pathogenesis. Firstly, the list of WT-circulating DEmiRNAs obtained from miRNA-Seq were subjected to the MIENTURNET web tool, and the miRTarBase (Huang et al., 2022) was selected for miRNA-target enrichment analysis. The following MIENTURNET settings were applied to limit our network: an FDR cutoff of 0.05, a minimum of five miRNA-target interactions, and strong experimental evidence.

### Statistical analysis

Statistical differences of dysregulated circulating miRNAs between experimental groups were evaluated by two-tailed t-test, with p-value <0.05 considered significantly dysregulated in WT. This cut-off was retained for our analyses. Further, Receiver-Operating Characteristic (ROC) analysis was performed using scikit learn library in the Python program to ascertain the diagnostic performance of analyzed miRNAs with a confidence interval of 95%. All statistical analysis was conducted in R studio (version 3.5.1). The Spearman Rank Correlation was used to evaluate the correlation between miRNA expression and clinicopathological variables related to WT.

## Results

### Clinicopathological data

All the subjects included in the study were from the Egyptian population, aged between 1 and 8 years, with a median diagnostic age of 3.5 years. Around 52% (14/27) of patients had FH-WTs, while 48% (13/27) had UnFH-WTs, with 3 having focal anaplasia and 10 identified as high-risk tumors with diffuse anaplasia (DAWTs). No congenital anomalies associated with WT were found in the study subjects. Approximately 15% (4/27) of patients had bilateral tumors, while the remaining cases had unilateral tumors with equal left and right-sided proportions. All WT patients were staged based on the COG pathological methodology, with 56% (15/27) of the children in clinical stage III, followed by 26% in stage IV (7/27), and 15% (4/27) in stage V. Among WT patients, 33% (9/27) had initial metastasis to the lung, while 67% (18/27) had non-metastatic tumors (Supplementary Table S1).

### NGS data analysis and read mapping

**FIGURE 1:**
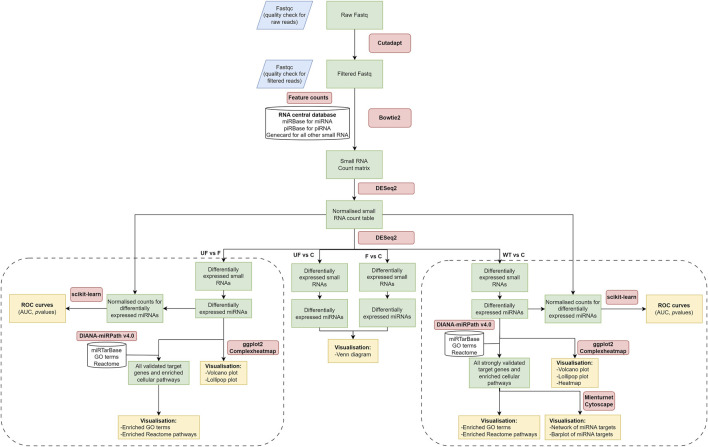
NGS data analysis workflow. It includes raw data quality check and filtering, adaptor trimming, alignment, miRNA quantification, and differential expression downstream analysis. The diagram was created using draw.io software.

Small RNA libraries were prepared, size-fractionated, and sequenced using NGS on the Illumina MiSeq sequencer. Raw reads were trimmed and filtered for quality control analysis, resulting in a median of 899,208 reads per sample (ranging from 0.7 to 1.5 million reads). NGS data analysis workflow is shown in (Figure 1). Mapping data to the RNA central database revealed that 14% of the total mapped reads were annotated as small ncRNAs. The distribution of the fraction of reads mapped to different ncRNAs were indicated in (Supplementary Figure S1; Supplementary Table S2). From the RNA central database, we obtained a comprehensive list of 1,007 annotated small ncRNAs, including 527 miRNAs commonly expressed in WT serum samples.

### A distinct expression pattern of circulating miRNAs in WT patients versus healthy controls

**FIGURE 2:**
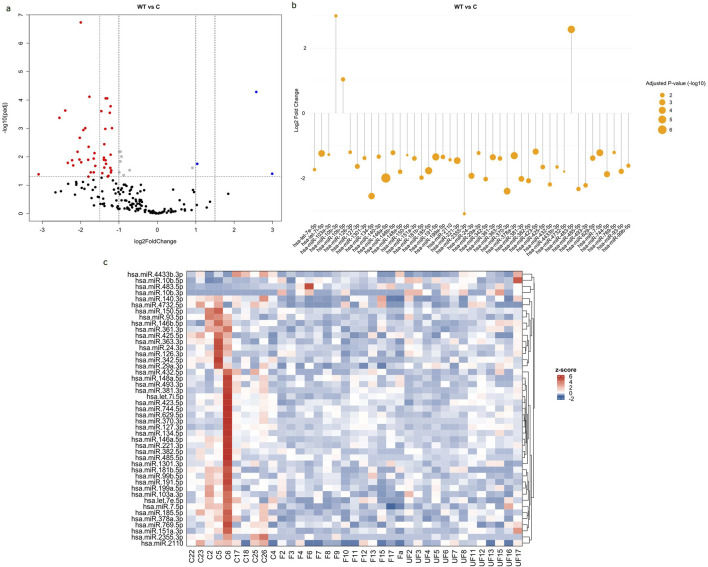
Differential expression of circulating miRNAs in WT serum samples compared to healthy controls. **(A)** Volcano plot of all expressed miRNA in serum samples, with log2 fold change (FC) plotted against-log10 (Adjusted p-value). Red dots indicate downregulated miRNAs with FC < -1 and p-value <0.05, while blue dots indicate upregulated miRNAs with FC > 1 and p-value <0.05. **(B)** Lollipop plot displays upregulated and downregulated DEmiRNAs based on log2fold change and adjusted p-value. The size of the orange circle is proportional to adjusted p-value. **(C)** Heat map displays the expression levels of miRNAs in WT samples and healthy controls. The labels “F” represents favorable tumors “UF” for unfavorable tumors and “C” for control samples. The red color represents upregulated miRNAs, whereas the blue color represents downregulated ones. Volcano and lollipop plots are visualized using ggplot2 R package, while heatmap is created using complexheatmap R package.

The study revealed significant differential expressions of 45 miRNAs in the serum of WT patients compared to healthy controls, with 3 upregulated and 42 downregulated. The values of all expressed miRNAs and their corresponding adjusted p-values were shown in a volcano plot (Figure 2A). The significant DEmiRNAs in WT serum samples were visualized in a lollipop plot (Figure 2B). The expression levels of DEmiRNAs between WT cases and healthy controls were visually represented in a Heat Map (Figure 2C).

An expression analysis of miRNAs in WT histopathological types compared to healthy controls revealed 51 significantly dysregulated miRNAs in FH-WTs (50 downregulated and 1 upregulated, hsa-miR-483-5p), and 28 significantly dysregulated miRNAs in the UnFH-WTs (25 downregulated and 3 upregulated) (Supplementary Table S3). There was a difference in the number of uniquely expressed miRNAs in each histopathological type, with 29 miRNAs exclusively dysregulated in FH-WTs and 6 miRNAs dysregulated solely in UnFH-WTs, as seen in (Supplementary Figure S2). In addition, 22 miRNAs were commonly expressed in both WT types.

Differentiating between WT histopathology types, hsa-miR-1180-3p showed significant differential expression between UnFH-WTs and FH-WTs, with high expression in UnFH-WT patients. All dysregulated miRNAs between WT and healthy controls are listed in (Supplementary Table S3) and the top DEmiRNAs are shown in Table 1.

**TABLE 1:** The top differentially expressed miRNAs in the serum of WT patients compared to healthy controls.

The top downregulated miRNAs in WT patients compared to healthy controls								
FH-WTs			UnFH-WTs			All WTs		
miRNA	log2 FC	adj p-value	miRNA	log2 FC	p-value	miRNA	log2 FC	adj p-value
miR-134-5p	−2.58	0.0018	miR-485-5p	−2.832	0.0244	miR-2355-3p	−3.094	0.0413
miR-20a-5p	−2.53	0.026	miR-370-3p	−2.600	0.0019	miR-134-5p	−2.550	0.0004
miR-342-5p	−2.47	0.008	miR-493-3p	−2.577	0.0255	miR-370-3p	−2.399	0.0002
miR-150-5p	−2.40	0.0017	miR-148a-5p	−2.515	0.0054	miR-485-5p	−2.332	0.0163
miR-24-3p	−2.31	0.0005	miR-134-5p	−2.508	0.0072	miR-493-3p	−2.220	0.0132
miR-370-3p	−2.23	0.002	miR-1180-3p	−2.286	0.0276	miR-432-5p	−2.191	0.0201
miR-382-5p	−2.15	0.0129	miR-99b-5p	−2.165	0.0115	miR-382-5p	−2.079	0.0066
miR-185-5p	−2.15	6.75E-06	miR-150-5p	−2.082	0.0148	miR-342-5p	−2.028	0.0124
miR-432-5p	−2.12	0.0497	miR-382-5p	−2.006	0.038	miR-381-3p	−2.020	0.0021
miR-181b-5p	−2.11	0.0260	miR-381-3p	−1.933	0.0174	miR-146a-5p	−1.994	1.85E-07
miR-381-3p	−2.10	0.0039	miR-146a-5p	−1.919	5.98E-05	miR-181b-5p	−1.983	0.0155
miR-146a-5p	−2.06	4.08E-06	miR-744-5p	−1.894	0.0084	miR-24-3p	−1.926	0.0011
miR-151a-3p	−1.99	0.0004	miR-93-5p	−1.716	0.0271	miR-744-5p	−1.877	0.0009
miR-101-3p	−1.96	0.0028	miR-425-5p	−1.695	0.0401	miR-148a-5p	−1.801	0.0127
miR-4433b-3p	−1.95	0.0280	miR-22-3p	−1.664	0.0072	miR-4732-5p	−1.795	0.0498
let-7e-5p	−1.95	0.0379	miR-24-3p	−1.601	0.0296	-miR-93-5p	−1.792	0.0044

The top upregulated miRNAs in WT patients compared to healthy controls								
FH-WTs			UnFH-WTs			All WTs		
miRNA	log2 FC	Adj p-value	miRNA	log2 FC	adj p-value	miRNA	log2 FC	adj p-value
miR-483-5p	2.8734	2.16E-05	miR-10a-5p	1.1448	0.0178	miR-10b-5p	1.0393	0.0176
			miR-10b-5p	1.3799	0.0072	miR-483-5p	2.5771	5.18E-05
			miR-483-5p	2.1709	0.006	miR-10b-3p	2.9918	0.0395

Differential expression of miRNAs between WT histopathology types				
MiRNA	log2 FC	adj p-value	Change	Comparison
hsa-miR-1180-3p	2.28678	0.027661	Up	UnFH-WT vs.FH-WT

### Pathway and functional enrichment analysis of circulating DEmiRNAs in WT

**FIGURE 3:**
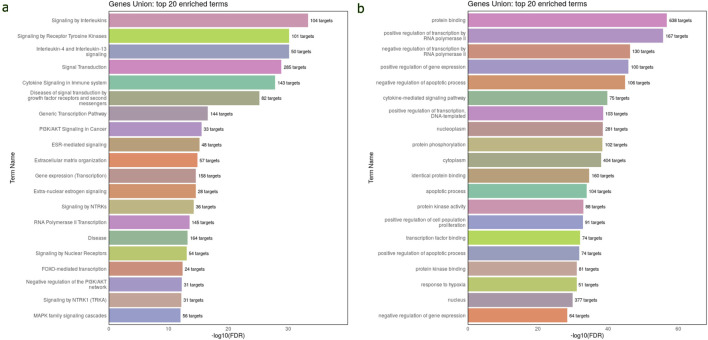
Reactome and GO enrichment analysis of validated target genes of 45 DEmiRNAs in the circulation of WT. These analyses are performed and visualized using the DIANA-miRPath v4.0 platform. **(A)** Bar chart displays the top 20 Reactome pathway affected by the dysregulated miRNAs in WT serum samples. **(B)** Bar chart displays the top 20 enriched GO terms affected by bloodstream dysregulated miRNAs in WT. The top pathways and GO terms are ranked based on the -log10(FDR), and the number of target genes involved in each pathway displayed next to every term.

The Reactome pathway analysis indicated that the target genes of 45 DEmiRNAs in WT are significantly enriched in the immune system signaling by interleukins and cytokines, suggesting a potential link between miRNA dysregulation and immune response modulation in WT development. Additionally, these genes are highly enriched in cancer-related pathways, such as PI3K/AKT, FOXO, and MAPK signaling pathways (Figure 3A). These pathways are essential for WT pathogenesis. The GO analysis revealed that DEmiRNAs in WT were involved in biological processes related to regulating transcription by RNA pol II, gene expression, apoptotic process, response to hypoxia and cell proliferation. These genes were also implicated in molecular functions, including protein binding, phosphorylation, and kinase activity. Regarding cellular component ontology, the validated target genes were primarily enriched in nucleoplasm, cytoplasm, nucleus, and cytosol (Figure 3B). The top 20 Reactome and GO enriched terms of DEmiRNAs in WT are listed in Table 2.

**TABLE 2:** The top 20 Reactome and GO enriched terms of target genes regulated by the DEmiRNAs in WT.

Reactome pathway	Term Genes	Target Genes (n)	miRNAs (n)	P-value	FDR
Signaling by Interleukins	512	104	29	2.20E-37	5.28E-34
Signaling by Receptor Tyrosine Kinases	528	101	28	6.38E-34	7.66E-31
Interleukin-4 and Interleukin-13 signaling	122	50	26	9.68E-34	7.75E-31
Signal Transduction	3,138	285	39	2.49E-32	1.50E-29
Cytokine Signaling in Immune system	1,050	143	32	3.36E-31	1.61E-28
Diseases of signal transduction by growth factor receptors and second messengers	415	82	28	1.68E-28	6.71E-26
Generic Transcription Pathway	1,372	144	35	8.41E-20	2.88E-17
PI3K/AKT Signaling in Cancer	103	33	23	1.01E-18	3.04E-16
ESR-mediated signaling	226	48	27	2.54E-18	6.77E-16
Extracellular matrix organization	318	57	21	6.77E-18	1.63E-15
Gene expression (Transcription)	1,661	158	35	1.27E-17	2.77E-15
Extra-nuclear estrogen signaling	78	28	22	1.46E-17	2.92E-15
Signaling by NTRKs	137	36	20	3.66E-17	6.76E-15
RNA Polymerase II Transcription	1,509	145	35	1.89E-16	3.24E-14
Disease	1,819	164	34	4.35E-16	6.97E-14
Signaling by Nuclear Receptors	318	54	28	6.04E-16	9.06E-14
FOXO-mediated transcription	67	24	20	3.26E-15	4.61E-13
Negative regulation of the PI3K/AKT network	117	31	18	4.62E-15	6.17E-13
Signaling by NTRK1 (TRKA)	118	31	19	5.99E-15	7.56E-13
MAPK family signaling cascades	360	56	24	8.91E-15	1.07E-12

Gene Ontology (GO)	Term Genes	Target Genes (n)	miRNAs (n)	P-value	FDR
protein binding	14,297	638	41	1.01E-61	1.90E-57
positive regulation of transcription by RNA polymerase II	1,258	167	33	2.27E-60	2.13E-56
negative regulation of transcription by RNA polymerase II	896	130	35	9.77E-51	6.11E-47
positive regulation of gene expression	519	100	34	3.41E-50	1.60E-46
negative regulation of apoptotic process	604	106	35	3.77E-49	1.41E-45
cytokine-mediated signaling pathway	319	75	27	4.88E-44	1.53E-40
positive regulation of transcription, DNA-templated	663	103	32	1.09E-42	2.92E-39
Nucleoplasm	4,107	281	37	1.45E-42	3.39E-39
protein phosphorylation	654	102	32	2.00E-42	4.16E-39
Cytoplasm	7,391	404	40	5.67E-42	1.06E-38
identical protein binding	1,679	160	32	1.46E-38	2.49E-35
apoptotic process	765	104	29	9.54E-38	1.49E-34
protein kinase activity	560	88	31	7.97E-37	1.15E-33
positive regulation of cell population proliferation	602	91	31	1.07E-36	1.43E-33
transcription factor binding	401	74	29	8.42E-36	1.05E-32
positive regulation of apoptotic process	404	74	27	1.42E-35	1.67E-32
protein kinase binding	501	81	33	8.10E-35	8.93E-32
response to hypoxia	178	51	23	9.63E-35	1.00E-31
Nucleus	7,177	377	39	1.47E-33	1.45E-30
negative regulation of gene expression	334	64	28	4.86E-32	4.55E-29

### The most promising miRNA-target interactions linked to WT

**FIGURE 4:**
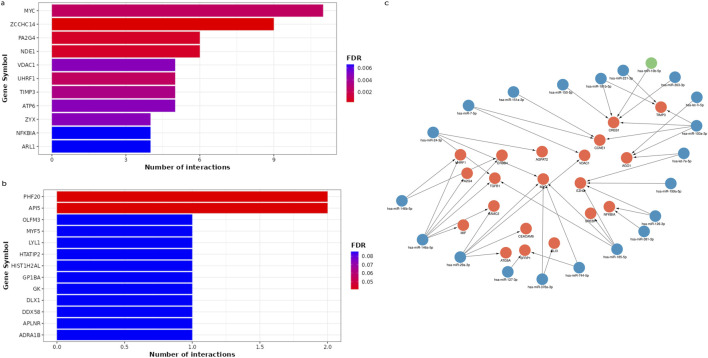
The miRNA–target enrichment analysis for circulating DEmiRNAs in WT patients using MIENTURNET tool. **(A)** Bar chart displays the top 11 target genes affected by downregulated miRNAs. The target genes were displayed on the horizontal axis, while the number of miRNA interactions was displayed on the vertical axis. Colors represent FDR values ranging from red to blue. **(B)** Bar plot represents the 11 most significant target genes affected by upregulated miRNAs. **(C)** A network for the strong validated miRNA-target interactions retrieved from miRTarbase using MIENTURNET tool and visualized using Cytoscape. The target genes are highlighted in red, while downregulated miRNAs are highlighted in blue, and upregulated miRNAs are highlighted in green.

The MIENTURNET results of miRNA-target enrichment analysis revealed that 42 downregulated miRNAs in WT were targeting 198 genes, with *ZCCHC14* and *MYC* having the highest number of miRNA interactions (Figure 4A). Three upregulated miRNAs targeted 8 genes, with *PHF20* and *API5* being the most significantly affected hub genes (Figure 4B). The network of the most promising miRNA-target interactions showed that several tumor suppressor and oncogenic miRNAs target and regulate critical genes, including *MYC, CREB1, TIMP3, CCNE1, and TGFB1*, which were shown to be involved in WT pathogenesis (Figure 4C).

### Correlation of circulating miRNA expressions with the advanced clinicopathological features of WT

The association between miRNA expression in WT serum samples and clinicopathological factors related to WT was assessed using the Spearman rank correlation test. The study findings indicated a significant positive correlation between the presence of initial metastasis and the decreased expression levels of hsa-miR-423-5p (*r* = 0.403, *p* = 0.036), hsa-miR-2355-3p (*r* = 0.439, *p* = 0.021), and hsa-miR-769–5 (*r* = 0.409, *p* = 0.034). Furthermore, there was a significant positive correlation between the lowered expression levels of hsa-miR-2355-3p (*r* = 0.6597, *p* = 0.0006) and hsa-miR-103a-3p (*r* = 0.4515, *p* = 0.0305) and the clinical stage of WT. The occurrence of bilateral kidney tumors was found to be positively correlated with lower levels of hsa-miR-342-5p (*r* = 0.3933, *p* = 0.04242). The relationship between miRNA expression and clinicopathological characteristics is shown in (Supplementary Table S4).

### Diagnostic potential of circulating DEmiRNAs in WT

**FIGURE 5:**
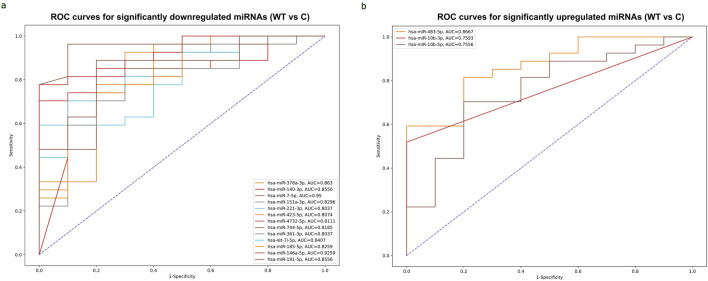
ROC curves of significantly differentially expressed miRNAs with the highest AUC values for detecting WT. **(A)** Combined ROC curves of 13 downregulated miRNAs for detecting WT. Vertical axis represents sensitivity and horizontal axis represents 1- specificity. Blue line represents reference and other colored lines represent downregulated miRNAs. **(B)** Combined ROC curves of 3 upregulated miRNAs for detecting WT. Sensitivity was represented on Vertical axis and horizontal axis represents 1- specificity. Blue line represent reference and other colored lines represents upregulated miRNAs. ROC curves are visualized using Python program (scikit learn library).

The ROC curve analysis demonstrated that, among miRNAs, 13 downregulated miRNAs and 3 upregulated miRNAs exhibited high AUC (Area under the ROC curve) values exceeding 0.8 and 0.7, respectively, indicating high specificity and sensitivity for WT detection. (Figures 5A, B). Among the 3 upregulated miRNAs, hsa-miR-483-5p showed the highest AUC value of 0.867, while out of the 13 downregulated miRNAs, hsa-miR-7-5p and hsa-miR-146a-5p demonstrated the highest AUC values of 0.95 and 0.926, respectively. These miRNAs may serve as potential biomarkers for WT detection due to their strong discriminatory power. ROC curves of significant upregulated and downregulated miRNAs with the highest AUC values were shown in (Supplementary Table S5).

### The potential of circulating hsa-miR-1180-3p to differentiate between anaplastic and non-anaplastic WT histopathology types and its functional enrichment

**FIGURE 6:**
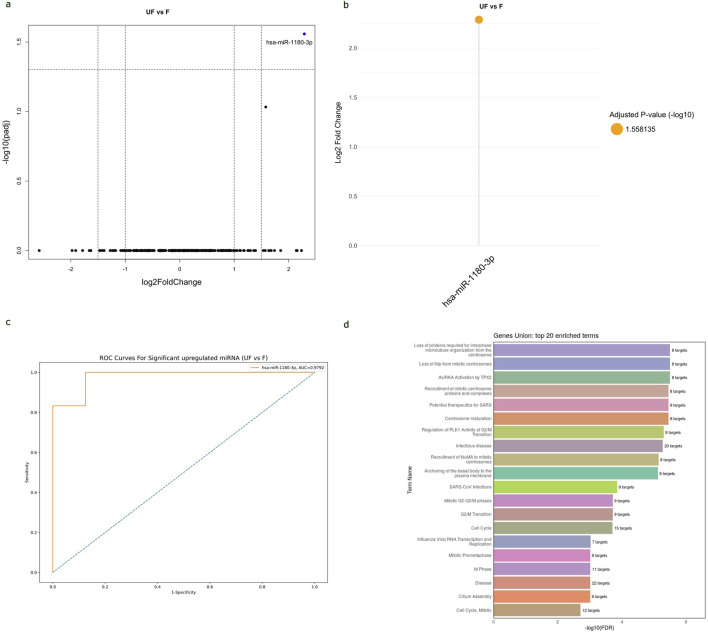
The potential of hsa-miR-1180-3p to distinguish between WT histopathology subtypes and its functional enrichment analysis. **(A)** Volcano plot of all miRNAs expressed in the serum samples of UnFH-WTs compared to FH-WTs, with log2 (fold change) plotted against-log10 adjusted p-value. Red dot indicates the overexpression of hsa-miR-1180-3p in UnFH-WTs. **(B)** Lollipop plot displays DEmiRNAs between UnFH-WTs and FH-WTs. In this case, hsa-miR-1180-3p (orange circle) showed significant upregulation in UnFH-WTs compared to FH-WT. Volcano and lollipop plots are visualized using ggplot2 R package **(C)** ROC curve for hsa-miR-1180-3p differentiating UnFH-WTs from FH-WTs. Vertical axis represents sensitivity and horizontal axis represents 1- specificity. Blue line represent reference and orange line represents hsa-miR-1180-3p. ROC curve is visualized using Python program (scikit learn library) **(D)**. Bar chart displays the top 20 Reactome enriched pathways of hsa-miR-1180-3p using the DIANA-miRPath v4.0. The enriched pathways are ranked based on the -log10 (FDR) and the number of target genes involved in each pathway displayed next to every term.

The study analyzed the differences in miRNA expressions and AUC values between FH-WT and UnFH-WTs to determine the potential of circulating miRNAs for distinguishing between WT histopathology types. The results have revealed that hsa-miR-1180-3p was the only miRNA that showed significant differential expression between the two groups. It is highly expressed in UnFH-WT patients compared to FH-WTs, with a log2 FC of 2.3 and an adjusted p-value of 0.027 (Figures 6A, B). The hsa-miR-1180-3p has also displayed high AUC value of 0.887, sensitivity of 92%, and specificity of 85% for detecting UnFH-WTs (Figure 6C), suggesting its potential as a non-invasive biomarker for differentiating between these two histology types of WTs.

The analysis of Reactome and GO enrichment revealed that hsa-miR-1180-3p target genes are highly enriched in pathways related to microtubule cytoskeleton organization, centrosome maturation, regulation of G2/M transition in cell cycle, and basal body-plasma membrane docking. These results suggest the potential role of hsa-miR-1180-3p in cell division, cell cycle and anaplasia progression in WT (Figure 6D). The top 20 Reactome and GO enriched terms of hsa-miR-1180-3p target genes are shown in (Supplementary Table S6).

## Discussion

Wilms’ tumor is a childhood kidney cancer that primarily affects children under 5 years old. Despite improvements in treatment and survival rates for patients with unilateral tumors or favorable histology, those with anaplastic features or bilateral tumors still exhibit poor prognosis and worse outcomes (Pater et al., 2021) Previous surveys have found abnormal miRNA expressions in WT, indicating their involvement in WT pathogenesis (Wang et al., 2010; Watson et al., 2013; Pérez-Linares et al., 2020).This study is the first to employ next-generation sequencing methodology to create a comprehensive circulating miRNA profile in WT and evaluate their potential as blood-based biomarkers to differentiate between favorable and unfavorable histology.

The circulating miRNAs in WT were previously reported, in some previous studies (Schmitt et al., 2012; Ludwig et al., 2015; Murray et al., 2015; Luo et al., 2020; Benlhachemi et al., 2023), conducted collectively on 280 samples of serum, plasma, and whole blood from 172 WT patients and 108 healthy controls using microarray and RT-PCR profiling assays. These previous studies (Schmitt et al., 2012; Ludwig et al., 2015; Murray et al., 2015; Luo et al., 2020; Benlhachemi et al., 2023) reported 301 dysregulated miRNAs, with 144 upregulated, 143 downregulated, and 14 inconsistent. While the present study identified 45 miRNAs with significant differential expressions in WT serum samples, 18 of which were previously reported and 27 were uniquely identified in this study (Supplementary Figure S3; Supplementary Table S7).

Out of 18 miRNAs that were previously detected by microarray and RT-PCR profiling assays (Supplementary Table S7), hsa-miR-483-5p, hsa-miR-10b-3p, and hsa-miR-10b-5, were found to be upregulated in WT patients’ serum, aligning with previous studies (Schmitt et al., 2012; Murray et al., 2015), indicating their oncogenic role in WT progression. Overexpression of miR-483-5p, a miRNA embedded within the *IGF2 gene*, has been linked to increased cell growth, invasion, and decreased cell death in WT cell lines (Liu et al., 2019). The co-expression pattern of miR-483-5p and *IGF2* indicates its involvement in WT tumorigenesis alongside *IGF2* (Ma et al., 2011). Furthermore, the miRNA-10 family has been found to induce Epithelial-to-Mesenchymal transition (EMT) through the PTEN/Akt pathway in renal fibrosis (Wang et al., 2022), indicating a possible link between these miRNAs and WT progression. Furthermore, our data has confirmed the significant downregulation of several circulating miRNAs, specifically hsa-miR-221-3p, hsa-miR-146a-5p, hsa-miR-378a-3p, hsa-miR-363-3p, hsa-miR-151a-3p, and hsa-miR-127-3p, which aligns with previous studies (Schmitt et al., 2012; Luo et al., 2020), suggesting their tumor-suppressive roles in WT.

The observed imbalance between upregulated and downregulated miRNAs in WTs aligns with prior research, indicating that miRNA biogenesis is frequently disrupted in cancer, leading to global miRNA suppression in tumor cells compared to normal tissue. Mutational analysis has shown that DROSHA and DICER1 mutations, commonly found in Wilms tumors, impair miRNA processing. Specifically, DROSHA mutations lead to global downregulation of miRNAs, while DICER1 mutations selectively disrupt the processing of 5′-arm miRNAs (Rakheja et al., 2014; Torrezan et al., 2014; Walz et al., 2015; Wegert et al., 2015). This suppression affects tumor-suppressing miRNAs, including members of the let-7 family. In our study, several let-7 miRNAs, such as miR-7-5p, let-7e-5p, let-7i-5p, let-7g-5p, and let-7a-5p, were notably downregulated. These disruptions in miRNA maturation may contribute to impaired kidney differentiation and tumorigenesis in WTs.

In the serum of WT patients, 27 miRNAs were uniquely identified in our study (Supplementary Table S7), among which, hsa-miR-2355-3p, hsa-miR-493-3p hsa-miR-134-5p, hsa-miR-370-3p, hsa-miR-485-5p, hsa-miR-432-5p, hsa-miR-382-5p, hsa-miR-342-5p, and hsa-miR-381-3p, exhibited significant downregulation with log2fold changes ≤ −2 in WT patients. Especially, hsa-miR-2355-3p exhibited the most substantial decrease in WT serum and demonstrated a significant positive correlation (p < 0.05) with clinical stage and initial metastasis in WT patients. These results verify that the lowered expression of hsa-miR-2355-3p is closely associated with advanced WT clinicopathological features, suggesting its potential as a biomarker for predicting the progression and severity of WT. Further investigation is needed to understand its mechanisms in WT development and metastasis. In other studies, on the other hand, miR-2355 levels were found to be increased in in the serum of lung adenocarcinoma (LUAD) patients and correlated with poor prognosis (Zhao et al., 2021). This suggests that the role of miR-2355 depends on the cancer subtype.

The number of uniquely expressed miRNAs was found to vary among histopathological WT types. Six miRNAs, namely, hsa-miR-485-5p, hsa-miR-493-3p, hsa-miR-148a-5p, hsa-miR-99b-5p, hsa-miR-10a-5p and hsa-miR-10b-5p, were found to be dysregulated only in UnFH-WTs, indicating their involvement in unfavorable WT progressionNotably, hsa-miR-485-5p, hsa-miR-493-3p and hsa-miR-148a-5p are newly identified in WT serum samples). A previous bioinformatic analysis of the TP53 signaling pathway in WT revealed that many target genes of hsa-miR-485-5p function as regulators of this pathway (He et al., 2020). Additionally, 80% of WTs with diffuse anaplasia have mutations in the *TP53* gene (Maschietto et al., 2014). These findings support the potential role of hsa-miR-485-5p in UnFH-WT development by affecting *TP53*. The hsa-miR-493-3p has already been reported as a tumor suppressor in various cancer types (Huang et al., 2019). The miR-148a downregulation has been found to be associated with inhibiting EMT and tumor growth in several malignancies (Li et al., 2016). The miR-99b-5p low expression in our study, contradicts previous study which showed elevated levels in WT serum samples (Murray et al., 2015). The inconsistency could be attributed to differences in patient populations, WT subtypes and detection assay. The high expression pattern of miR-10a in nephron progenitors was found to correlate with an early decline in nephron progenitors, increased apoptosis, and premature disruption of nephrogenesis (Ho et al., 2011). This finding suggests that miR-10a may contribute to WT pathogenesis by disrupting normal nephrogenesis process.

The Reactome pathway analysis has shown that the circulating DEmiRNAs in WT are significantly enriched in various cancer-related pathways, such as the PI3K/AKT, FOXO, and MAPK signaling pathways. These pathways have been recently investigated in WT pathogenesis. The PI3K-AKT-p53 axis has been found to promote WT tumorigenesis (Zhang et al., 2016). The FOXO signaling pathway is crucial for various cellular functions, such as DNA damage repair, apoptosis, and oxidative stress (Eijkelenboom and Burgering, 2013). *FOXO3a,* a tumor suppressor gene, has been observed to inhibit growth and invasion of nephroblastoma cells by disrupting the Wnt/β-catenin signaling pathway (Geng et al., 2022).The MAPK pathway has been found to be involved kidney growth, branching of the Ureteric Bud, and nephric duct connection to the cloaca. It is also involved in nephrogenesis control (Kurtzeborn et al., 2019). The GO analysis has revealed that circulating DEmiRNAs in WT target critical genes involved in biological processes such as apoptosis, gene expression, and transcription. These genes, including *TP53, IGF1R, IGF1, WNT1, MMP9, VEGFA, BCL2, BMP4, PTEN, NOTCH2, and FAS*, have been implicated in tumor progression.

The miRNA-target interaction network showed that several tumor suppressor miRNAs identified in our study, such as hsa-miR-146a-5p, hsa-miR-378a-3p, hsa-miR-29a-3p and hsa-miR-185-5p, target multiple critical genes, including *MYC, CREB1, TIMP3, CCNE1, and TGFB1*, which were also shown to be involved in WT pathogenesis.

The study found significant positive correlations (p < 0.05) between miRNA expression and clinical and pathological characteristics in WTs. Reduced expression levels of hsa-miR-423-5p, hsa-miR-2355-3p, and hsa-miR-769-5p were significantly associated with initial metastasis, while decreased levels of hsa-miR-2355-3p and hsa-miR-103a-3p were positively correlated with clinical stage. The incidence of bilateral kidney tumors was associated with lower levels of hsa-miR-342-5p. Overall, these findings highlight the potential role of specific miRNAs in predicting the progression and metastasis of WT. In previous studies, these miRNAs have been found to play significant roles in the progression of various cancers, such as breast cancer, lung adenocarcinoma, colorectal cancer, and cervical cancer (Dai et al., 2020; Zhao et al., 2021; Xian et al., 2019; Ren et al., 2022), promoting cell proliferation, migration, and invasion and correlated with adverse clinicopathological characteristics related to these cancers.

The ROC analysis has indicated that three upregulated miRNAs, hsa-miR-10b-3p, hsa-miR-10b-5p and hsa-miR-483-5p, have high diagnostic potential for WT, consistent with previous study (Murray et al., 2015) which demonstrated high sensitivity and specificity rates of these miRNAs. The hsa-miR-483-5p has been linked to chemoresistance and poor prognosis in WT tissues, indicating its potential as a prognostic marker (Watson et al., 2013). Additionally, 13 downregulated miRNAs showed high diagnostic performance for WT, with hsa-miR-7-5p and hsa-miR-146a-5p showing the highest AUC values exceeding 0.9, indicating their potential as non-invasive biomarkers for WT diagnosis. The hsa-miR-146a-5p was proposed as a biomarker for distinguishing between WT and diffuse hyperplastic perilobar nephroblastomatosis (DHPLN) due to its downregulation during malignization (Csók et al., 2023).

This study assessed the potential of DEmiRNAs to differentiate between favorable and unfavorable WT subtypes two major histopathology types of WT. The circulating hsa-miR-1180-3p showed a significant differential expression with a 2.3 log2 fold difference in expression betweenUnFH-WTs and FH-WTs. This miRNA also displayed high sensitivity (92%) and specificity (85%) rates for detecting UnFH-WTs, suggesting its potential as a blood-based biomarker for identifying poor prognostic subtypes of WT. A previous study revealed that upregulation of miR-1180-5p in WT tissues positively correlates with histological type, stage, and lymph node metastasis. The inhibition of miR-1180 reduced WT growth and induced apoptosis *in vivo* by targeting p73 (Jiang and Li, 2018). This miRNA may function as an oncogene or tumor suppressor in many tumors (Hu et al., 2019; Li et al., 2021). The miR-1180-3p overexpression has been found to enhance cell division and glycolysis in ovarian cancer cells through the SFRP1/Wnt signaling pathway (Hu et al., 2019). Moreover, it stimulates cell growth, migration, and infiltration in lung cancer through the Wnt/β-catenin and PI3K/AKT pathways (Chen et al., 2017). MiR-1180-3p has also been identified as a potential diagnostic indicator for multiple cancers, including melanoma and gastric cancer (Zhu et al., 2019; Guo et al., 2021).

The Reactome pathway analysis of miR-1180-3p revealed that its target genes are highly enriched in pathways related to essential cellular processes, such as cell division and cell cycle regulation. Dysregulation of these pathways can lead to abnormal cell proliferation and tumorigenesis, highlighting the potential importance of miR-1180-3p in the development of diffuse anaplasia and unfavorable histological features in WT. Further validation is needed to confirm its regulatory mechanisms in WT signaling pathways.

## Conclusion

The study has identified a distinct signature of 45 DEmiRNAs in the serum of WT patients compared to healthy controls, with 27 miRNAs that were newly detected. The hsa-miR-7-5p, hsa-miR-146a-5p, hsa-miR-378a-3p, and hsa-miR-483-5p have shown the highest discriminatory power, indicating their potential as non-invasive biomarkers for WT diagnosis. The expression of hsa-miR-2355-3p has not been previously identified in WT serum and its decreased expression has been significantly linked to advanced WT clinicopathological features, suggesting its potential as a biomarker for predicting WT progression and severity. The significant upregulation of hsa-miR-1180-3p in UnFH-WT patients could serve as a reliable blood-based biomarker for distinguishing between favorable and unfavorableWT subtypes. Further research with a larger sample size is warranted to validate these findings and explore potential therapeutic targets for unfavorable WTs. Investigating the mechanisms by which hsa-miR-1180-3p regulate cell division and cell cycle could provide a deeper understanding of its role in WT pathogenesis.

## Data Availability

The datasets presented in this study can be found in online repositories. The names of the repository/repositories and accession number(s) can be found below: https://www.ncbi.nlm.nih.gov/, SRA repository, BioProject database ID PRJNA1112148.

## Glossary

API5: apoptosis inhibitor 5
BCL2: BCL2 apoptosis regulator
BMP4: bone morphogenetic protein 4
CCNE1: cyclin E1
COG: Children Oncology Group
CREB1: cAMP responsive element binding protein 1
CTNNB1: Catenin Beta 1
DEmiRNAs: Differentially expressed miRNAs
DGCR8: DiGeorge syndrome critical region 8
DHPLN: diffuse hyperplastic perilobar nephroblastomatosis
EMT: Epithelial-to-Mesenchymal transition
FAS: Fas cell surface death receptor
FDR: False Discovery rate
**FH**-**WTs**: Favorable histology Wilms tumor)
FOXO: The mammalian forkhead transcription factors of the O class
GO: Gene Ontology
GRCh38: Human Reference Genome
ICH GCP: International Council for Harmonisation of Technical Requirements for Pharmaceuticals for Human Use (ICH) Guideline for Good Clinical Practice (GCP)
IGF1: insulin like growth factor 1
IGF1R: insulin like growth factor 1 receptor
IGF2: insulin like growth factor 2
IRB: Institutional Review Board
LIN28: Lin-28 homolog A
LUAD: lung adenocarcinoma
MAPK: Mitogen-activated protein kinases
MIENTURNET: MIcroRNA Enrichment TURned NETwork
MMP9: matrix metallopeptidase 9
NCBI: National Center for Biotechnology Information
NGS: Next-generation sequencing
NOTCH2: notch receptor 2
PHF20: PHD Finger Protein 20
PI3K: phosphoinositide-3-kinase
pol II: RNA polymerase II
PTEN: phosphatase and tensin homolog
**qRT**-PCR: Quantitative Real-Time Polymerase Chain Reaction
ROC: Receiver-Operating Characteristic curve analysis
SFRP1: secreted frizzled related protein 1
SMAC: Scientific Medical Advisory Committee
SRA: Sequence Read Archive data
STM: Renal Study Team Meeting
TGFB1: transforming growth factor beta 1
TIMP3: TIMP metallopeptidase inhibitor 3
TLDA: TaqMan Low-Density Array
TP53: Tumor protein P53
UnFH-WTs: Unfavorable histology Wilms tumor
VEGFA: vascular endothelial growth factor A
WNT1: Wnt family member 1
WT: Wilms tumor
WT1: Wilms Tumor Gene 1
WTX: Wilms Tumor Gene on X Chromosome
ZCCHC14: Zinc Finger CCHC-Type Containing 14
