# Supplementary material for: Profiling of the serum MiRNAome in pediatric egyptian patients with wilms tumor
Source: Front Mol Biosci. 2024 Oct 15;11:1453562. doi: 10.3389/fmolb.2024.1453562 (PMC11519528; doi:10.3389/fmolb.2024.1453562)
Supplement: Supplementary file 1 [file DataSheet1.PDF]

Supplementary Material

Profiling of the Serum MiRNAome in Pediatric Egyptian Patients with Wilms Tumor

Fatma S. Mohamed, Deena Jalal, Youssef M. Fadel, Samir F. El-Mashtoly, Wael Z. Khaled, Ahmed A. Sayed*, Mohamed A Ghazy
*** Correspondence:** Ahmed A. Sayed Ahmed.Sayed@57357.org

1 Supplementary Figures and Tables

1.1 Supplementary Figures

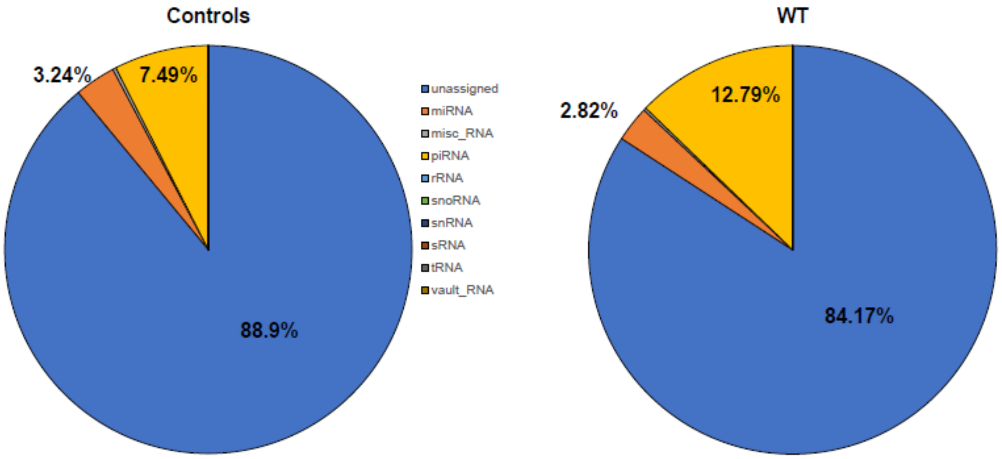

Supplementary Figure 1. Percentages of aligned reads to different small ncRNAs in both WT and healthy control samples

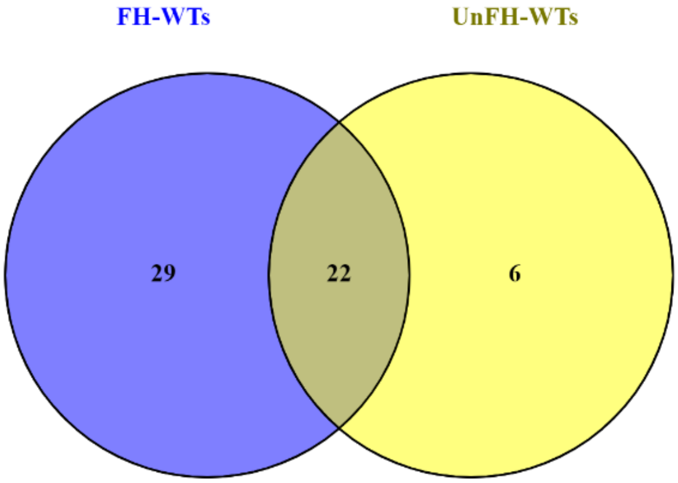

Supplementary Figure 2. A venn diagram of the common and unique dysregulated miRNAs between UnFH-WTs and FH-WTs. The diagram was visualized using Venny tool v 2.1.

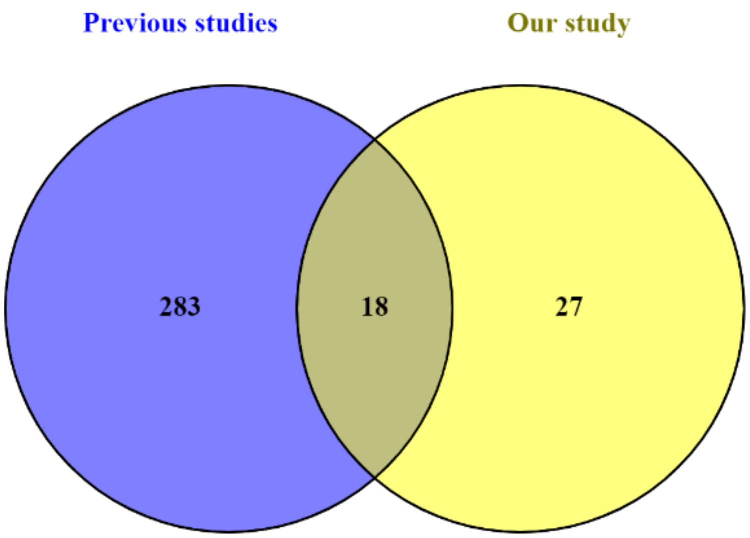

Supplementary Figure 3. AVenn diagram of the common and unique DEmiRNAs identified between the present and previous studies in the circulation of WT patients. The diagram was visualized using Venny tool v 2.1.0

1.2 Supplementary Tables

Supplementary Table 1. Clinicopathological Characteristics of WT patients and heathy individuals

**Characteristics**	**Wilms Tumor cases (n=27)**	**Healthy controls cases (n=10)**
**Diagnostic Age**	(Median 3.5yrs)	(Median 3.5yrs)
**Gender**		
Female	14	6
Male	13	4
**Tumor Histology**		
FH-WT	14	
UnFH-WT	13	
**Stage**		
II/III	16	
IV	7	
V	4	
**Laterality**		
Unilateral tumor	16	
Bilateral Tumor	11	
**Metastasis**		
Present	9 (lung)	
Absent	18	

Supplementary Table 2. Small RNA Sequencing Data and Mapped Reads

Samples	Total Read counts After Trimming	Aligned Reads	Reads mapped to miRNAs	Reads mapped to piRNAs
Control Samples (n=10)	10,001,017	1,102,221	324,990	749,388
WT Samples (n=27)	22,200,168	3,514,684	626,992	2,839,991
All Samples (n=37)	32,201,185	4,616,905	951,982	3,589,379

Supplementary Table 3. All differentially expressed miRNAs in WT serum samples compared to healthy controls.

The downregulated miRNAs in WT Patients compared to healthy controls								
FH-WTs			UnFH-WTs			All WTs		
miRNA	log2 FC	adj p-value	miRNA	log2 FC	adj p-value	miRNA	log2 FC	adj p-value
miR-134-5p	-2.58	0.001	miR-485-5p	-2.83	0.024	miR-2355-3p	-3.09	0.0413
miR-20a-5p	-2.53	0.026	miR-370-3p	-2.60	0.001	miR-134-5p	-2.55	0.000
miR-342-5p	-2.47	0.008	miR-493-3p	-2.57	0.025	miR-370-3p	-2.39	0.000
miR-150-5p	-2.40	0.001	miR-148a-5p	-2.51	0.005	miR-485-5p	-2.33	0.016
miR-24-3p	-2.31	0.000	miR-134-5p	-2.50	0.007	miR-493-3p	-2.22	0.013
miR-370-3p	-2.23	0.002	miR-1180-3p	-2.28	0.027	miR-432-5p	-2.19	0.020
miR-382-5p	-2.15	0.012	miR-99b-5p	-2.16	0.011	miR-382-5p	-2.07	0.006
miR-185-5p	-2.15	0.000	miR-150-5p	-2.08	0.014	miR-342-5p	-2.02	0.012
miR-432-5p	-2.12	0.049	miR-382-5p	-2.00	0.038	miR-381-3p	-2.02	0.002
miR-181b-5p	-2.11	0.026	miR-381-3p	-1.93	0.017	miR-146a-5p	-1.99	0.000
miR-381-3p	-2.10	0.003	miR-146a-5p	-1.91	0.000	miR-181b-5p	-1.98	0.015
miR-146a-5p	-2.06	0.000	miR-744-5p	-1.89	0.008	miR-24-3p	-1.92	0.001
miR-151a-3p	-1.99	0.000	miR-93-5p	-1.71	0.027	miR-744-5p	-1.87	0.000
miR-101-3p	-1.96	0.002	miR-425-5p	-1.69	0.04	miR-148a-5p	-1.80	0.012
miR-4433b-3p	-1.95	0.028	miR-22-3p	-1.66	0.007	miR-4732-5p	-1.79	0.049
let-7e-5p	-1.95	0.037	miR-24-3p	-1.60	0.029	miR-93-5p	-1.79	0.004
miR-29a-3p	-1.92	0.000	miR-127-3p	-1.51	0.045	miR-185-5p	-1.76	0.000
miR-363-3p	-1.90	0.001	miR-185-5p	-1.45	0.007	let-7e-5p	-1.73	0.035
miR-93-5p	-1.86	0.008	miR-629-5p	-1.42	0.014	miR-425-5p	-1.65	0.011
miR-744-5p	-1.86	0.003	miR-221-3p	-1.33	0.008	miR-4433b-3p	-1.65	0.035
miR-127-3p	-1.76	0.008	miR-191-5p	-1.31	0.003	miR-127-3p	-1.63	0.007
miR-1180-3p	-1.76	0.016	miR-378a-3p	-1.27	0.003	miR-99b-5p	-1.61	0.021
miR-2110	-1.67	0.035	miR-181a-5p	-1.24	0.038	miR-221-3p	-1.45	0.000
miR-425-5p	-1.61	0.031	miR-361-3p	-1.22	0.017	miR-2110	-1.43	0.037

miR-22-3p	-1.59	0.003	miR-423-5p	-1.14	0.009	miR-363-3p	-1.39	0.012
miR-221-3p	-1.58	0.000	has-let-7i-5p	-1.12	0.008	miR-151a-3p	-1.39	0.011
miR-146b-5p	-1.56	0.001				miR-629-5p	-1.38	0.003
miR-199a-3p	-1.55	0.019				miR-1301-3p	-1.38	0.023
miR-7-5p	-1.53	0.000				miR-361-3p	-1.35	0.001
miR-103a-3p	-1.52	0.034				miR-199a-5p	-1.34	0.013
miR-361-3p	-1.48	0.001				miR-191-5p	-1.34	0.000
miR-140-3p	-1.48	0.019				miR-140-3p	-1.33	0.018
miR-199a-5p	-1.47	0.016				miR-378a-3p	-1.30	0.000
miR-769-5p	-1.44	0.026				miR-150-5p	-1.29	0.049
miR-126-3p	-1.37	0.026				miR-103a-3p	-1.27	0.046
miR-191-5p	-1.37	0.0004				let-7i-5p	-1.23	0.000
miR-181a-5p	-1.37	0.010				miR-29a-3p	-1.22	0.024
let-7i-5p	-1.35	0.000				miR-7-5p	-1.21	0.000
miR-378a-3p	-1.34	0.000				miR-146b-5p	-1.21	0.008
miR-629-5p	-1.34	0.010				miR-769-5p	-1.20	0.035
miR-486-5p	-1.29	0.019				miR-126-3p	-1.20	0.03
miR-16-2-3p	-1.25	0.020				miR-423-5p	-1.18	0.0009
miR-30d-5p	-1.23	0.001						
miR-423-5p	-1.21	0.002						
miR-186-5p	-1.20	0.001						
miR-21-5p	-1.15	0.026						
let-7g-5p	-1.15	0.007						
miR-451a	-1.11	0.049						
miR-25-3p	-1.08	0.049						
let-7a-5p	-1.04	0.008						
**The upregulated miRNAs in WT serum samples compared to healthy controls.**								
**FH-WTs**			**UnFH-WTs**			**All WTs**		
miRNA	log2 FC	adj p-value	miRNA	log2 FC	adj p-value	miRNA	log2 FC	adj p-value
miR-483-5p	2.87	0.000	miR-10a-5p	1.14	0.017	miR-10b-5p	1.039	0.017
			miR-10b-5p	1.37	0.007	miR-483-5p	2.577	0.000
			miR-483-5p	2.17	0.006	miR-10b-3p	2.991	0.039

Supplementary Table 4. The highest AUC values obtained from ROC analysis of significant dysregulated miRNAs in WT compared to healthy controls

miRNA NAME	AUC	P-value	Change	Comparison
hsa-miR-7-5p	0.95	0.0001	Down	WT vs Control
hsa-miR-146a-5p	0.926	0.0001	Down	WT vs Control
hsa-miR-378a-3p	0.863	0.001	Down	WT vs Control
hsa-miR-140-3p	0.856	0.001	Down	WT vs Control
hsa-miR-191-5p	0.856	0.001	Down	WT vs Control
hsa-let-7i-5p	0.841	0.002	Down	WT vs Control
hsa-miR-151a-3p	0.83	0.002	Down	WT vs Control
hsa-miR-185-5p	0.826	0.003	Down	WT vs Control
hsa-miR-744-5p	0.819	0.003	Down	WT vs Control
hsa-miR-4732-5p	0.811	0.004	Down	WT vs Control
hsa-miR-423-5p	0.807	0.005	Down	WT vs Control
hsa-miR-221-3p	0.804	0.005	Down	WT vs Control
hsa-miR-361-3p	0.804	0.005	Down	WT vs Control
hsa-miR-483-5p	0.867	0.001	UP	WT vs Control
hsa-miR-10b-3p	0.759	0.017	UP	WT vs Control
hsa-miR-10b-5p	0.756	0.018	UP	WT vs Control

Supplementary Table 5. The top 20 Reactome and GO enriched terms of hsa-miRNA-1180-3p target genes

Reactome Pathway	Term Genes	Target Genes (n)	P-value	FDR
AURKA Activation by TPX2	84	8	3.96E-09	3.17E-06
Loss of Nlp from mitotic centrosomes	81	8	2.95E-09	3.17E-06
Loss of proteins required for interphase microtubule organization from the centrosome	81	8	2.95E-09	3.17E-06
Potential therapeutics for SARS	91	8	7.52E-09	3.58E-06
Centrosome maturation	93	8	8.95E-09	3.58E-06
Recruitment of mitotic centrosome proteins and complexes	93	8	8.95E-09	3.58E-06
Regulation of PLK1 Activity at G2/M Transition	99	8	1.47E-08	5.05E-06
Infectious disease	1054	20	1.78E-08	5.34E-06
Recruitment of NuMA to mitotic centrosomes	107	8	2.73E-08	7.28E-06
Anchoring of the basal body to the plasma membrane	109	8	3.16E-08	7.58E-06
SARS-CoV Infections	161	8	6.49E-07	0.000142
G2/M Transition	232	9	9.83E-07	0.000195

Mitotic G2-G2/M phases	234	9	1.06E-06	0.000195
Cell Cycle	764	15	1.15E-06	0.000198
Influenza Viral RNA Transcription and Replication	153	7	5.96E-06	0.000953
Disease	1819	22	7.53E-06	0.00097
Mitotic Prometaphase	224	8	7.67E-06	0.00097
M Phase	462	11	6.59E-06	0.00097
Cilium Assembly	222	8	7.18E-06	0.00097
Cell Cycle, Mitotic	612	12	1.70E-05	0.001941
**Gene Ontology (GO)**	**Term Genes**	**Target Genes (n)**	**P-value**	**FDR**
nuclear envelope lumen	18	8	9.51E-16	1.78E-11
cellular process	21	8	4.38E-15	4.11E-11
cytoskeleton-dependent intracellular transport	26	8	3.32E-14	2.07E-10
natural killer cell mediated cytotoxicity	31	8	1.65E-13	7.73E-10
RNA binding	1707	29	7.07E-13	2.65E-09
cell body	102	10	3.92E-12	1.22E-08
microtubule-based process	47	8	6.28E-12	1.68E-08
MHC class I protein binding	56	8	2.76E-11	6.47E-08
cytoplasmic ribonucleoprotein granule	94	9	6.12E-11	1.27E-07
azurophil granule lumen	105	9	1.67E-10	3.14E-07
structural constituent of cytoskeleton	112	9	3.00E-10	5.11E-07
regulation of G2/M transition of mitotic cell cycle	93	8	1.78E-09	2.77E-06
ubiquitin protein ligase binding	335	12	3.28E-09	4.73E-06
Nucleus	7177	52	5.49E-09	6.86E-06
ciliary basal body-plasma membrane docking	107	8	5.45E-09	6.86E-06
G2/M transition of mitotic cell cycle	150	8	7.75E-08	9.08E-05
microtubule cytoskeleton organization	160	8	1.28E-07	0.000141
mitotic cell cycle	165	8	1.62E-07	0.000168
cytoskeleton organization	183	8	3.57E-07	0.000353
extracellular exosome	2384	25	7.33E-07	0.000687

Supplementary Table 6. The common and unique WT circulating miRNAs identified in the current research.

The common dysregulated miRNAs between the present and previous studies						
**miRNA**	**Sample**	**Cases**	**Profiling Assay**	**change in previous studies**	**change in our study**	**Reference**
hsa-miR-423-5p<br>hsa-miR-483-5p	whole blood	41unilateral<br>2 bilateral	Microarrays	Up<br>Up	Down<br>Up	Schmitt et al, 2012 doi.org/10.1186/1471-2164-13-379
hsa-miR-126-3p<br>hsa-miR-181b-5p<br>hsa-miR-24-3p	serum	9 blastemal<br>11regressive<br>12 triphasic<br>7 metastatic	RT-qPCR	UP<br>UP<br>UP	Down<br>Down<br>Down	Ludwig et al, 2015 doi.org/10.1002/pbc.25481
hsa-miR-10b-3p<br>hsa-miR-99b-5p	serum	6 unilateral<br>1 bilateral	RT-qPCR	UP<br>UP	UP<br>Down	Murray et al, 2015 doi.org/10.1158/1055-9965.EPI-14-0669
hsa-miR-221-3p<br>hsa-miR-103a-3p<br>hsa-miR-191-5p<br>hsa-miR-146a-5p<br>hsa-miR-150-5p<br>hsa-miR-185-5p<br>hsa-miR-363-3p<br>hsa-miR-378a-3p<br>hsa-miR-151a-3p<br>hsa-miR-425-5p<br>hsa-miR-127-3p	whole blood	45 FH<br>15 UnFH	Microarrays	Down<br>Up<br>Up<br>Down<br>Up<br>Up<br>Down<br>Down<br>Down<br>Up<br>Down	Down<br>Down<br>Down<br>Down<br>Down<br>Down<br>Down<br>Down<br>Down<br>Down<br>Down	Luo et al, 2020 doi.org/10.1016/j.biopha.2020.109880

The new circulating miRNAs identified in the present study					
**miRNA**	**log2 FC**	**Adj p-value**	**miRNA**	**log2 FC**	**Adj p-value**
hsa-miR-2355-3p	-3.094	0.0413	hsa-miR-4433b-3p	-1.65	0.0352
hsa-miR-134-5p	-2.550	0.0004	hsa-miR-2110	-1.433	0.0376
hsa-miR-370-3p	-2.399	0.0002	hsa-miR-629-5p	-1.382	0.0035
hsa-miR-485-5p	-2.332	0.0163	hsa-miR-1301-3p	-1.381	0.0239
hsa-miR-493-3p	-2.220	0.0132	hsa-miR-361-3p	-1.355	0.0012

hsa-miR-432-5p	-2.191	0.0201	hsa-miR-199a-5p	-1.347	0.0132
hsa-miR-382-5p	-2.079	0.006	hsa-miR-140-3p	-1.337	0.0170
hsa-miR-342-5p	-2.028	0.0124	hsa-let-7i-5p	-1.238	0.0002
hsa-miR-381-3p	-2.020	0.0021	hsa-miR-29a-3p	-1.224	0.0245
hsa-miR-744-5p	-1.877	0.0009	hsa-miR-7-5p	-1.218	0.0001
hsa-miR-148a-5p	-1.801	0.0127	hsa-miR-146b-5p	-1.216	0.0084
hsa-miR-4732-5p	-1.795	0.0498	hsa-miR-769-5p	-1.209	0.0352
hsa-miR-93-5p	-1.790	0.0044	hsa-miR-10b-5p	1.0393	0.0176
hsa-let-7e-5p	-1.735	0.03529			
